# Recombinant Sialyltransferase Infusion Mitigates Infection-Driven Acute Lung Inflammation

**DOI:** 10.3389/fimmu.2019.00048

**Published:** 2019-02-04

**Authors:** Mehrab Nasirikenari, Amit A. Lugade, Sriram Neelamegham, Zhongwei Gao, Kelley W. Moremen, Paul N. Bogner, Yasmin Thanavala, Joseph T. Y. Lau

**Affiliations:** ^1^Department of Molecular and Cellular Biology, Roswell Park Comprehensive Cancer Center, Buffalo, NY, United States; ^2^Department of Immunology, Roswell Park Comprehensive Cancer Center, Buffalo, NY, United States; ^3^Department of Chemical and Biomedical Engineering, University at Buffalo, Buffalo, NY, United States; ^4^The Complex Carbohydrate Research Center, University of Georgia, Athens, GA, United States; ^5^Department of Pathology, Roswell Park Comprehensive Cancer Center, Buffalo, NY, United States

**Keywords:** sialylation, ST6Gal-1, inflammation, infection, airway, extrinsic glycosylation

## Abstract

Inappropriate inflammation exacerbates a vast array of chronic and acute conditions with severe health risks. In certain situations, such as acute sepsis, traditional therapies may be inadequate in preventing severe organ damage or death. We have previously shown cell surface glycan modification by the circulating sialyltransferase ST6Gal-1 regulates *de novo* inflammatory cell production via a novel extrinsic glycosylation pathway. Here, we show that therapeutic administration of recombinant, bioactive ST6Gal-1 (rST6G) mitigates acute inflammation in a murine model mimicking acute exacerbations experienced by patients with chronic obstructive pulmonary disease (COPD). In addition to suppressing proximal neutrophil recruitment at onset of infection-mediated inflammation, rST6G also muted local cytokine production. Histologically, exposure with NTHI, a bacterium associated with COPD exacerbations, in rST6G-treated animals revealed consistent and pronounced reduction of pulmonary inflammation, characterized by smaller inflammatory cuffs around bronchovascular bundles, and fewer inflammatory cells within alveolar walls, alveolar spaces, and on pleural surfaces. Taken together, the data advance the idea that manipulating circulatory ST6Gal-1 levels has potential in managing inflammatory conditions by leveraging the combined approaches of controlling new inflammatory cell production and dampening the inflammation mediator cascade.

## Introduction

Acute inflammation is protective and intrinsic to a healing process. However, dysregulated, excessive, or persistent inflammation is detrimental and is often implicated in chronic conditions including cardiovascular, respiratory, and rheumatic diseases, and in extreme cases, systemic inflammatory response syndromes with high risks for mortality. Previously we provided evidence that a glycan-modifying enzyme present in systemic circulation is a potent regulator of inflammatory cell production (1–3). This enzyme, the ST6Gal-1 sialyltransferase, is regarded as a resident of the Golgi-ER secretory network, mediating the attachment of α (2, 4)-linked sialic acid residues to exposed lactosaminyl-bearing nascent glycoproteins during intracellular biosynthetic transit. However, there is a significant pool of extracellular ST6Gal-1, particularly in the blood (5). It has been known for a number of decades that changes in the level of circulatory ST6Gal-1 and the circulatory sialyl-glycan structures constructed by ST6Gal-1 are associated with a diverse array of clinical conditions including stress (6), atherosclerosis (4, 7), alcoholism (8, 9), as well as certain cancers, particularly colon and breast cancers, and multiple myeloma (10–12). Studies in the 1980's have established that elevated release of ST6Gal-1 into the blood was a component of the hepatic acute phase response (13, 14). Within the last decade, there has been a renewed interest implicating ST6Gal-1 expression in chemoresistance (15), TNF and EGF-mediated signal transduction (16, 17), maintenance of pluripotentency in stem cells (18, 19), and cancer (10, 20, 21). The renewed interest has been based on the assumption of cell-autonomously expressed enzyme, and insight into the functional relevance of ST6Gal-1 released into the blood has remained relatively overlooked.

In a departure from the canonical mode of Golgi-ER glycosylation, which is a cell-autonomous and intracellular process, the extracellular, blood ST6Gal-1 remodels glycans on target cell surfaces in a novel extrinsic mechanism, which is not cell-autonomous (22, 23). Two genetically modified mouse models were used in these studies. The first, *St6gal1*-KO, was globally ST6Gal-1 deficient (24). The other, *St6gal1*-dP1, was deficient only in the liver-derived extracellular pool of ST6Gal-1 in the blood (25). Comparative analysis of these models revealed an overly robust inflammation and exaggerated inflammatory cell production associated with ST6Gal-1 deficiency. Exaggerated inflammation was attributed to deficiency only in the circulating extracellular pool, and not in the intracellular secretory apparatus-bound enzyme (1–3, 5). Lack of circulating ST6Gal-1 resulted in an exaggerated neutrophilic peritonitis upon challenge with *Salmonella typhimurium* or with the sterile eliciting agent, thioglycollate (2, 25). Circulatory ST6Gal-1 deficiency also resulted in more acute Th2 pulmonary inflammation with excessive eosinophil infiltration and elevated inflammatory cytokine release in OVA-sensitized mice (3). Recently, we observed that systemic ST6Gal-1 modifies the Granulocyte-Monocyte Progenitor (GMP) subset of hematopoietic progenitors, attenuating the production of granulocytes by blunting the transition of GMPs into Granulocyte Progenitors (1), thus providing a mechanistic explanation of how insufficiency in the blood-borne pool of ST6Gal-1 promotes a generally pro-inflammatory condition with excessive granulocyte production. We recently showed that subcutaneous implantation of localized B16 melanoma engineered to overexpress ST6Gal-1 could partially alleviate neutrophilic airway inflammation when challenged intratracheally with LPS in mice (1). Extracellular, systemic ST6Gal-1 was identified recently to be a pro-survival factor in transitional B cell development in the marrow, supporting a concept that circulating ST6Gal-1 is a conveyor of systemic cues guiding the development of multiple branches of immune cells (26).

In the present report, we tested the hypothesis that elevating blood ST6Gal-1 activity, by directly infusing a recombinant form of ST6Gal-1 (rST6G), can have therapeutic value in dampening inflammation. Lung diseases such as Chronic Obstructive Pulmonary Disease (COPD), the 4th leading cause of death worldwide, are characterized by episodic bouts of acute inflammation. These acute exacerbations, triggered by bacterial and viral infections, allergens, or other noxious stimuli, lead to an influx of inflammatory immune cells, predominantly granulocytes and macrophages, which drive disease pathology (27, 28). In the most severe forms, these episodes of immune cell recruitment can be directly life threatening, and at best they promote long-term airway destruction leading to permanently diminished airway functions. We used a murine model of acute airway inflammation elicited by NTHI (Non-typable *Haemophilus influenza*), an opportunistic pathogen common in acute exacerbations of COPD (27, 28). Repeated exposure of mice to NTHI recapitulated many of the features of airway damage seen in human COPD including induction and persistence of perivascular lymphocytic infiltrates and tissue destruction where the initial influx of inflammatory cells is thought to contribute centrally to drive organ damage in later stages (29). We observed that animals receiving rST6G 2 h after an NTHI instillation had strikingly less acute inflammation with reduced pathology and less neutrophil infiltration into the lung, when compared to animals receiving only saline. Furthermore, rST6G treated animals had notably blunted local release of inflammatory cytokines. *Ex vivo* treatment of airway macrophages with rST6G resulted in muted NTHI –dependent production of inflammatory mediators. The data point to the value of rST6G administration in alleviating inflammation by suppressing new inflammatory cell production and in mitigating excessive inflammation by blunting the release of inflammatory cytokines.

## Results

### Reduced Circulatory ST6Gal-1 Is Associated With More Severe Acute Airway Inflammation

**Figure 1:**
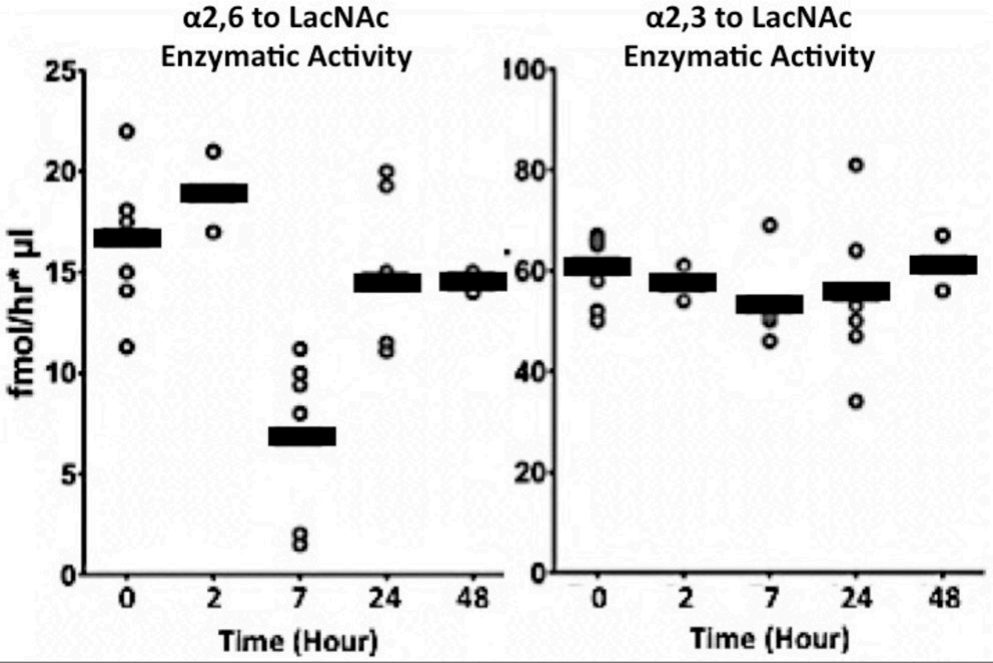
Transient depression of circulatory ST6Gal-1 accompanies acute airway inflammation. Live NTHI bacteria (10^6^ CFU / animal) were delivered by oropharengeal instillation. Blood was collected at the times shown after instillation. Sialyltransferase activities in the sera were measured by following the transfer of CMP-[3H]Sia to Galβ1–4GlcNAc-O-Bn (LacNAc). The Siaα2,6 product formed by ST6Gal-1 **(Left)**, was separated from Siaα2,3 product formed by various ST3Gal transferases **(Right)** using SNA-agarose chromatography.

To validate that there is an inverse relationship between the naturally occurring ST6Gal-1 in circulation and the need to produce new inflammatory cells during demand granulopoiesis, we subjected naïve, native C57BL/6 mice to a challenge with NTHI directly into the airways. NTHI elicits a Type 1 immune response in the airways that is dominated by neutrophilic infiltration in the initial phase. Circulatory ST6Gal-1 was monitored in these animals by assessing the enzymatic ability to form α2,6-sialyl linkages onto Gal(β1,4)GlcNAc acceptor substrate. NTHI exposure generated a pronounced but transient depression of circulatory ST6Gal-1 activity to ~30% of baseline levels at 7 h (Figure 1, left). In contrast, other sialyltransferase activities in the blood, specifically those forming the α2,3-sialyl structures on Gal(β1,4)GlcNAc and mediated by the sialyltransferases ST3Gal-3,−4, or−6, were not altered upon NTHI exposure (Figure 1, right).

**Figure 2:**
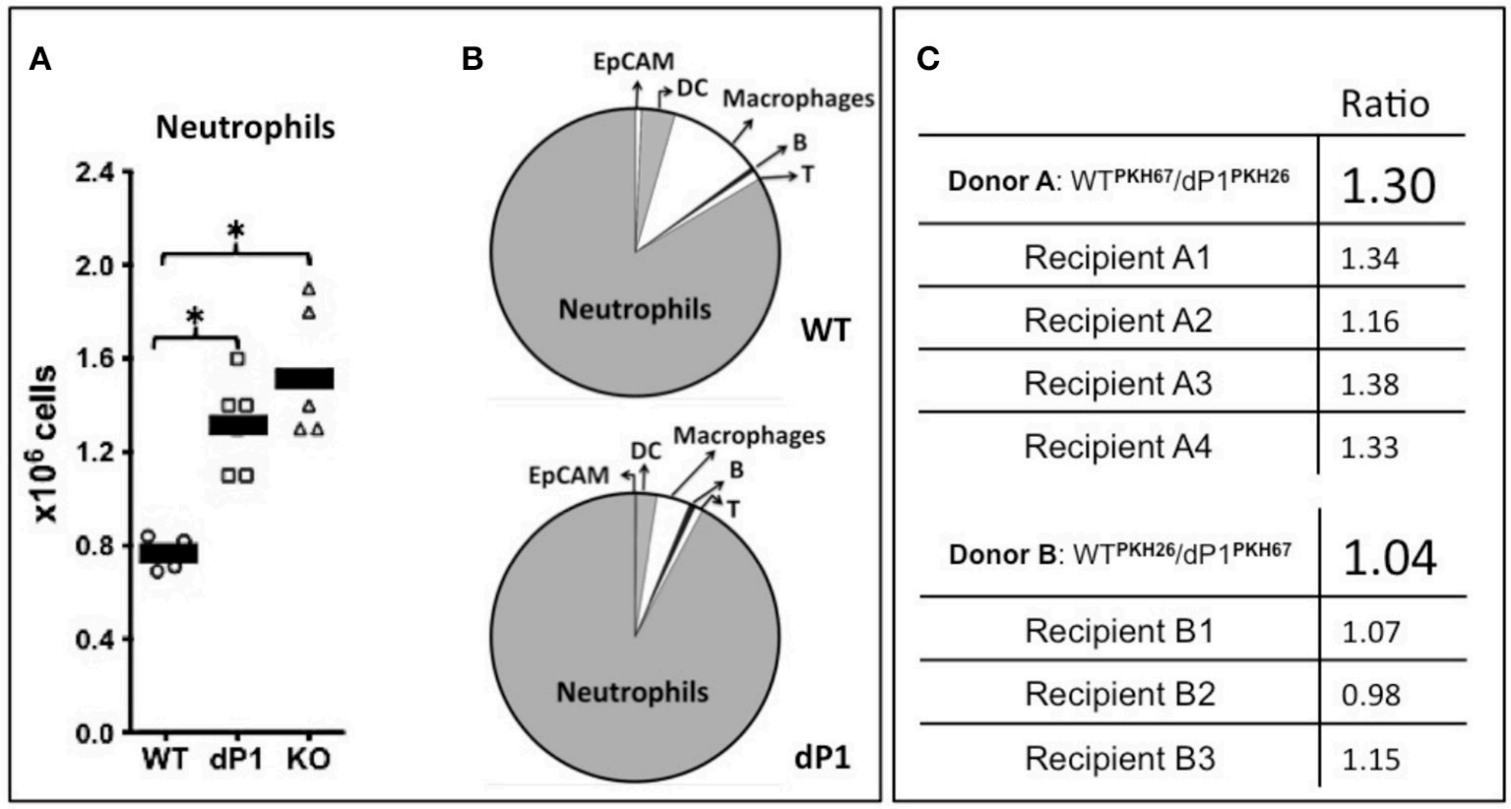
More severe neutrophilic acute airway inflammation in animals with ST6Gal-1 deficiency. Wild type C57BL/6 (WT), *ST6Gal1-dP1* (dP1), and *ST6Gal1-KO* (KO) mice were exposed to 10^6^ CFU of live NTHI bacteria by oropharengeal instillation. Eighteen hours later, the bronchoalveolar lavage fluid (BALF) was collected, Leukocyte number was counted, and leukocyte composition was determined by flow cytometry. **(A)** shows the total numbers of neutrophils recovered from the BALF of NTHI-instilled animals, showing greater neutrophilic inflammation in dP1 and KO, compared to WT (1.6 and 2.0-fold, respectively). ^*^ *p* < 0.05 for indicated comparisons. **(B)**, top shows the cellular composition of WT BALF, consisting predominantly of neutrophils (83.5%). Macrophage (10.5%), dendritic cells (DC, 3.8%), T- (1%), and B- (0.5%) cells, with a minor constituent of epithelial cells as defined by EpCAM (0.7%). **(B)**, bottom, shows dP1 BALF composition, which was essentially identical to WT BALF in percentage cellular contribution from the assessed cell types. **(C)** Neutrophils from the marrows of WT and dP1 mice were isolated by negative selection. The cells were stained with one of the two distinct membrane dyes (red PKH-26 and green PKH-67), mixed in an ~1:1 ratio and injected into 3–4 WT recipients 2 h after NTHI challenge. In the top panel, the initial PKH67-labeled WT/ PKH26-labeled dP1 neutrophils ratio was 1.30. In the bottom panel, PKH26-WT/PKH67-dP1 neutrophil donor ratio was 1.04. At 18 h, this neutrophil-fluorescence ratio was again measured in cells obtained from the BALF. No difference in airway recruitment was noted for dP1 neutrophils compared to WT neutrophils.

We reported previously that insufficient circulatory ST6Gal-1 levels result in accelerated *de novo* granulocyte accumulation (1–3). Here, we validated this observation in the NTHI model of acute airway inflammation. The globally ST6Gal-1 null mouse, *St6gal1*-KO, and the St6gal1-dP1 mouse with deficiency only in the circulatory pool of extracellular ST6Gal-1 were examined. As summarized in Figure 2A, both *St6gal1*-dP1 and *St6gal1*-KO mice had exaggerated neutrophil accumulation in the BALF compared to wild-type animals 18 h after NTHI challenge. Moreover, nearly identical ~1.5-fold augmented accumulation of neutrophils was observed in both ST6Gal-1 deficit models that were generated independently by different strategies. Neutrophils dominated the inflammatory cell infiltrate into the airway in the acute inflammatory response to NTHI. On average, 0.8 × 10^6^ neutrophils were recoverable in the bronchial alveolar lavage fluid (BALF) at 18 h in native C57BL/6 (wild-type) animal, and neutrophils comprised >75% of the total recovered BALF cells (Figure 2B). This result, which is more fully presented in Supplemental Table 1A, strongly supports the conclusion that insufficiency in circulatory ST6Gal-1, rather than intracellular Golgi-ER bound ST6Gal-1, was the factor driving excess neutrophil accumulation in the airways in acute inflammation.

Enhanced efficiency in recruitment of granulocytes into the airway of *St6gal1*-dP1 mice compared to wild-type animals did not drive the exaggerated neutrophil accumulation. Neutrophils were isolated from *St6gal1*-dP1 and C57BL/6 mice, differentially labeled with either PKH26 or PKH67 fluorescent dyes, pooled, and intravenously transferred into wild-type recipients. The recruitment of the adoptively infused neutrophils into NTHI-induced acute airway was monitored as ratios of the differentially dyed cells by flow cytometry. Figure 2C shows that the ratios of the *St6gal1*-dP1 to wild-type neutrophils recovered in the BALF were not changed from the original input ratios. The data confirm that circulatory ST6Gal-1 deficiency resulted in a more severe acute airway inflammatory response to NTHI challenge, and that the mechanism is more robust granulopoiesis rather than altered efficiencies of cell recruitment to the inflamed lung.

### Direct Intravenous Infusion of Recombinant ST6Gal-1 Mitigated Acute Airway Inflammation

**Figure 3:**
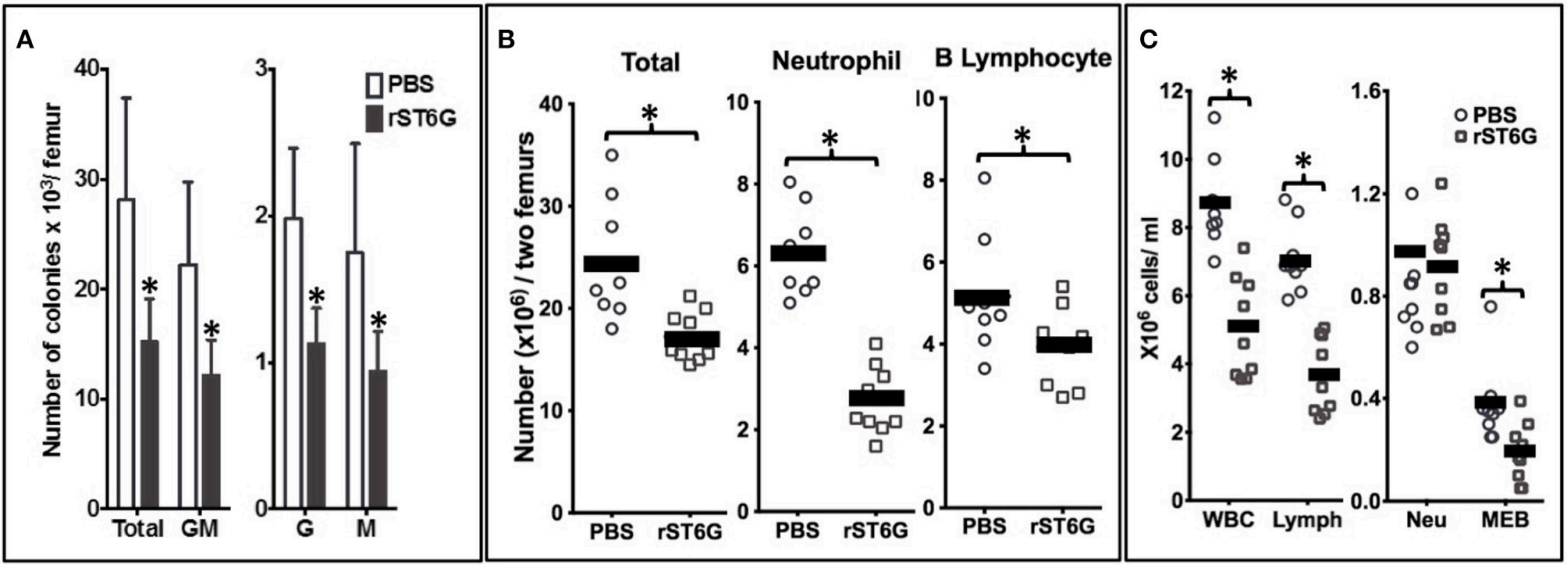
Intravenous rST6G infusion depresses myelopoiesis and alters inflammatory cell availability. WT mice receiving either a single 300 μg bolus of recombinant ST6Gal-1 (rST6G) or saline (PBS) were sacrificed 7 h later. Bone marrow cells from hind limbs and peripheral blood were isolated and analyzed as follows. **(A)** presents marrow progenitor clonogenic activity for granulocyte/monocyte (GM), granulocyte (G), or monocyte (M) progenitor colonies. The combined total colony formed is also shown (Total). Saline- (open bars) and rST6G-treated mice (*n* = 9 each group) were used with 4 × 10^4^ marrow cells were plated in Methocult M3534 to promote growth of myeloid progenitors for 10 days. ^*^p < *0.0*1 rST6G compared to PBS. **(B)** summarizes the overall bone marrow cellularity of PBS (round symbols) and rST6G-treated (square symbols) animals, where each symbol denotes one animal. Total bone marrow cellularity (Total), and neutrophil and B cell numbers are shown. ^*^p < *0.01*. **(C)** Summarizes white cell counts in the blood as total white blood cell (WBC) count and differential count for lymphocyte (Lymph), neutrophil (Neu), monocyte, eosinophil and basophil (MEB). PBS (*n* = 8) or rST6G-treated animals (*n* = 9) were used. ^*^p < *0.01*.

We observed in murine genetic models of circulatory ST6Gal-1 deficiency more pronounced peritonitis and airway acute inflammation elicited by sterile agents such as LPS (1, 22). Chronic elevation of circulatory ST6Gal-1 by subcutaneous implantation of a B16 melanoma engineered to release ST6Gal-1 partially alleviated the sterile agent induced acute airway (1). Here, we posit that an infection-driven acute inflammation can also be attenuated by raising circulatory ST6Gal-1. We further posit that infusion of pure, recombinant ST6Gal-1 (rST6G), resulting only in temporary elevation of blood ST6Gal-1 activity can be effective against infection-driven inflammation. To explore the potential therapeutic value of rST6G, this and all other following experiments were performed in the wild-type C57BL/6 mouse. A single bolus of rST6G in its present formulation, when infused into wild-type animals at baseline was rapidly cleared from the bloodstream in <1 h (see Supplemental Figure 1A). Despite the rapid clearance, a single rST6G infusion resulted in a striking decline in granulopoietic parameters within the bone marrow, with ~40% decrease in colony forming units in granulocyte (G), Monocyte (M) and GM-CFUs 7 h later (Figure 3A). Total marrow cellularity diminished overall by ~25%, resulting mostly from a >2-fold reduction in marrow neutrophils and a slight reduction in B220-positive cells, the two most-abundant marrow cell populations (Figure 3B). Blood differentials revealed an almost 50% reduction in total white cell counts, accountable by the diminution of circulatory lymphocyte numbers that are the major white cell constituents in the blood (Figure 3C). Curiously, circulatory neutrophils in the naïve wild-type animals were not altered, although at baseline only a minor (10–12%) percentage of the overall circulating white cells are granulocytes. The complete blood differential counts are presented in Supplementary Table 1B.

**Figure 4:**
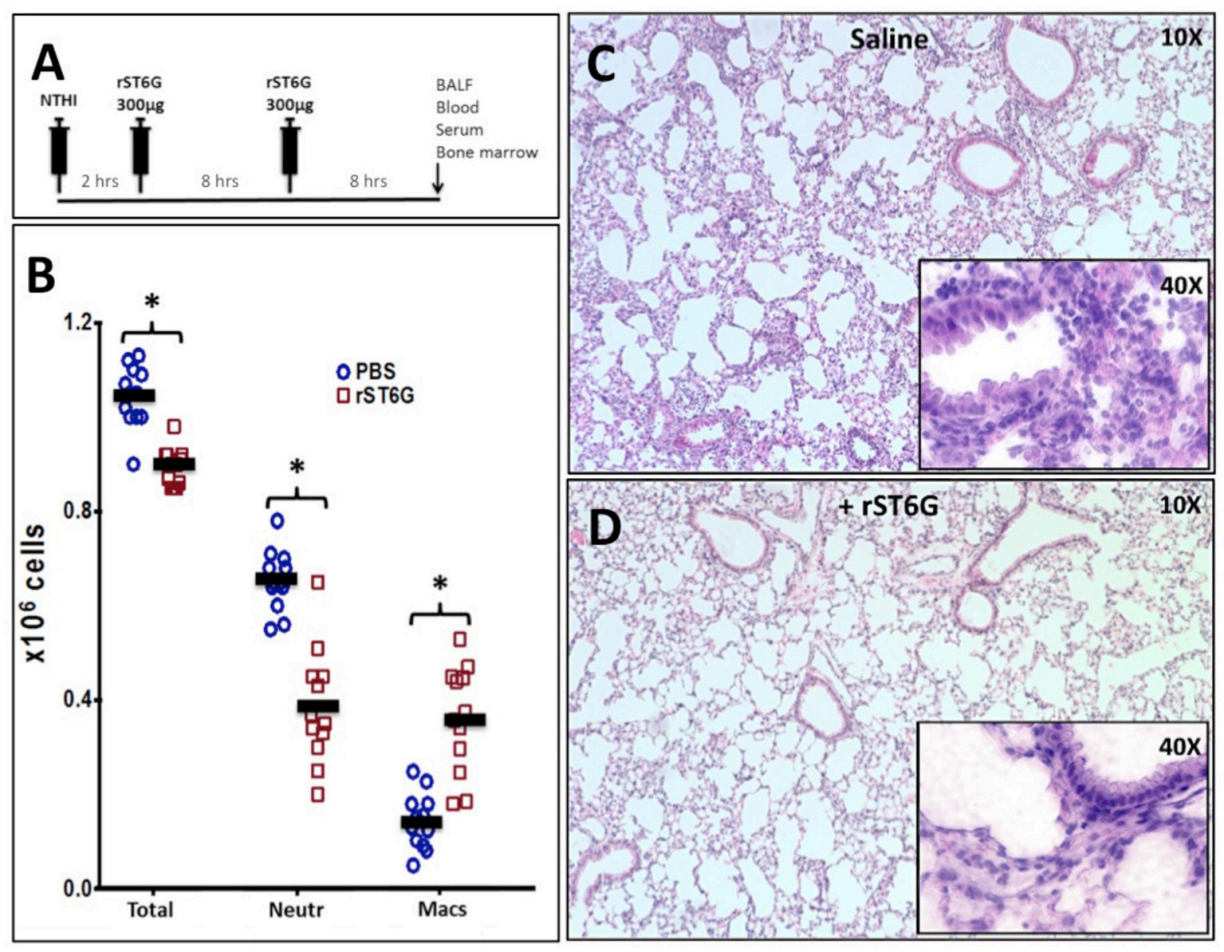
Acute airway inflammation induced by NTHI exposure is mitigated by rST6G infusion. **(A)** shows the intervention protocol where each animal received two 300 μg rST6G or saline/sham injections spaced 8 h apart with the first injection being at 2 h after NTHI challenge. Animals were sacrificed 18 h later and assessed for pulmonary inflammation. **(B)** Inflammatory cell accumulation in the BALF of the rST6G- and sham- (PBS) treated animals showing total BALF cells (Total), neutrophil (Neutr), and macrophage accumulation (Macs). ^*^p < 0.01. **(C,D)** Show the lung pathology of saline and rST6G-treated animals, respectively at 18 h.

**Figure 5:**
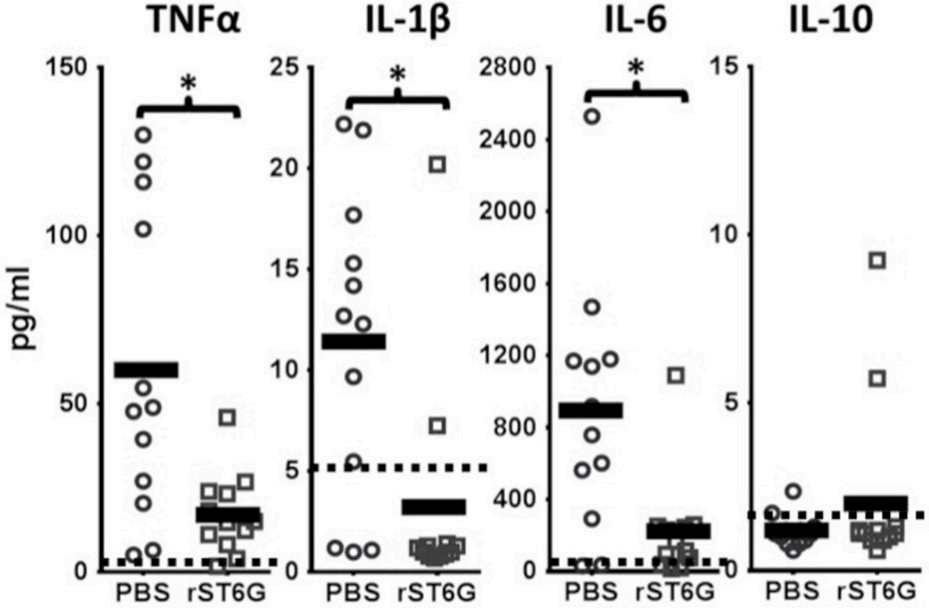
Inflammatory cytokine release during acute airway inflammation is suppressed by rST6G infusion. C57BL/6 wild-type animals were challenged with NTHI and subjected to the rST6G or sham (PBS) treatment protocol as outlined in Figure 4A. The bronchial alveolar lavage fluids (BALF) were analyzed for cytokines by Luminex 100 multiplex assays. Dashed lines and the shaded boxed regions represent the reliable lower assay limit of detection for each cytokine. Many of the values obtained, especially for the IL-10 assays and the rST6G-treated cohorts for IL-1β values, were zero. For these, a default low value of “1” was assigned in order to calculate the *p* value. ^*^p < 0.001.

To assess the anti-inflammatory efficacy of systemically administered rST6G, NTHI-challenged C57BL/6 mice received 2 intravenous infusions of rST6G, the first at 2 h after receiving NTHI, and a booster at 10 h. Two intravenous rST6G infusions were used as a precaution, because we observed rapid clearance of the current formulation of rST6G from the blood (Supplemental Figure 1A). The two infusions 5, 8 h apart resulted in circulatory ST6Gal-1 activity that was 2-fold over baseline at 16h after the initial rST6G infusion (Supplemental Figure 1B). In cohorts receiving rST6G, BALF neutrophil counts were reduced by ~50% (Figure 4B). Only a slight (10%) reduction in overall BALF leukocyte counts was observed, due to a ~2.5-fold increase in recruited macrophage. Though the increase in recruited macrophage numbers was unexpected, it is noteworthy that animals with genetic ST6Gal-1 deficit had ~33% decrease in recruited macrophage numbers in the airway following NTHI exposure (see Supplemental Table 1A). Circulating blood counts, monitored at the time BALF was recovered, showed no differences between rST6G and sham animals. This is not unexpected, since circulating neutrophilia was noted to be extremely transient and limited to the first few hours after a peripheral acute challenge, including peritonitis by LPS or thioglycollate (2), airway eosinophilia by OVA to sensitized mice (3), and acute airway inflammation by LPS (30). Blinded histopathologic evaluation disclosed a consistent reduction in pulmonary inflammation among animals treated with rST6G. Compared to animals receiving saline, the rST6G treated group showed smaller inflammatory cuffs around bronchovascular bundles and fewer inflammatory cells within alveolar walls and alveolar spaces (Figures 4C,D). The histopathologic scoring is summarized in Supplemental Table 2. Most unexpectedly, the rST6G-treated group had strikingly lowered levels of inflammatory cytokines TNF-α, IL-1β, and IL-6 in the BALF. In fact, inflammatory cytokines were close to or below reliable assay detection limits in BALF from animals that received rST6G, when compared to easily quantifiable levels in sham treated animals (Figure 5).

**Figure 6:**
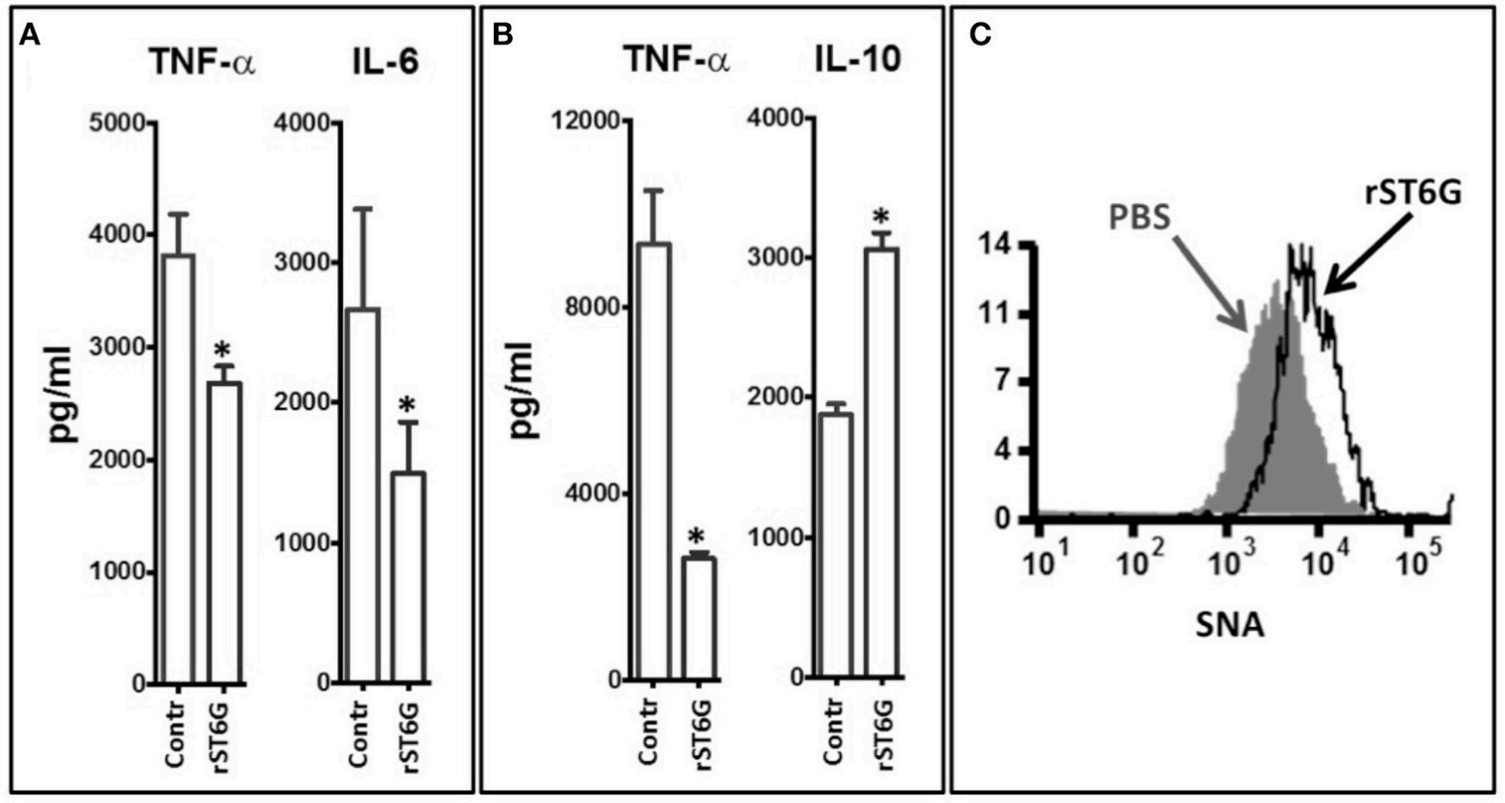
Inflammatory cytokine release by macrophage was attenuated by ST6Gal-1. **(A)** Macrophages were recovered from the BALF of 3 wild-type C57BL/6 mice (at rest). The pooled cells were plated into 5 replicate but identical wells for each determination. Groups of 5 macrophage wells were exposed *ex vivo* to 10^5^ CFU/ml heat-killed NTHI either in the absence or presence (20 μg/ml) of rST6G and CMP-Sia (100 μM) for 18 h. TNF-α and IL-6 released into the media was measured next day. ^*^p < 0.001. **(B)** Bone marrow–derived macrophages were generated from marrow cells of C57BL/6 WT animals. The identically seeded cells, in groups of 5 wells per treatment, were exposed to heat-killed NTHI in the absence (control) or presence of rST6G (20 μg/ml) and CMP-Sia (100 μM) and incubated overnight (37°C and 5% CO_2_). TNF-α and IL-10 released into the media were assessed by ELISA. ^*^p < 0.001 based on concentration values from five separate wells for each condition. All data are representative of six separate experiments. **(C)** BALF macrophage was recovered from C57BL/6 WT animals 18 h after oropharyngeal challenge with NTHI using the protocol in Figure 4A, either with rST6G (rST6G) or sham (PBS). Cell surface sialylation was measured using the α2,6-sialic acid-specific lectin, FITC conjugated *Sambucus nigra* agglutinin (SNA) by flow cytometry, as shown.

To gain mechanistic insight into the blunted inflammatory cytokines released in the airways by rST6G treatment, despite the apparently paradoxical elevation of airway macrophage, one of the principal cell types along with epithelial cells responsible for the release of inflammatory cytokines (31, 32), we examined the response of primary airway macrophages. Airway macrophages isolated from the BALF of resting wild-type C56BL/6 mice were stimulated *ex vivo* with heat-killed NTHI in the presence or absence of rST6G. A reduction of NTHI-dependent release of TNF-α and IL-6 production was observed in the rST6G-treated macrophages (Figure 6A). This effect was not unique to airway macrophages, as bone marrow derived macrophages treated with rST6G also had a 3.5-fold reduction in TNF-α (Figure 6B), Interestingly, IL-10 production by NTHI stimulated BM macrophages was elevated in the rST6G treated cells, pointing tantalizingly to a possible additional pathway by which rST6G can mitigate acute inflammation. In these *ex vivo* experiments, 0.1 mM CMP-Sia was also included. We have observed that rST6G to have effect on suppressing macrophage activity *ex vivo*, even without added sialic acid donor substrate. Possibly, the leakage of sialic acid donor substrate from neighboring dying cells might be sufficient. However, we also observed that addition of CMP-Sia has the benefit of diminishing variability. *In vivo*, sialic acid donor substrate is believed to be supplied by activating platelets (23, 33).

In an earlier report, we showed that systemic ST6Gal-1 dampens granulopoiesis in the marrow by extrinsic modification of the hematopoietic progenitor cells through the attachment of α2,6-linked sialic acid residues, which can be monitored by the lectin, SNA (*Sambucus nigra* agglutinin) (22). Here, cell surface α2,6-sialylation status of airway macrophage recovered in the BALF of animals challenged *in vivo* with NTHI was assessed for changes in SNA reactivity. The data show a pronounced increase in cell surface SNA reactivity in cells from animals treated with rST6G, compared to saline treated animals (Figure 6C). This observation strongly suggests that rST6G introduced into systemic circulation were able to affect the pulmonary macrophages and blunt the release of inflammatory cytokines during an NTHI-elicited acute airway response.

## Discussion

It has long been known that changes in the level of circulatory ST6Gal-1 and the circulatory sialyl-glycan structures constructed by ST6Gal-1 are associated with a diverse array of clinical conditions including stress (6), atherosclerosis(4, 34), alcoholism (35, 36), as well as in a certain cancers, particularly colon, breast cancers and multiple myeloma (10–12). Studies in the 1980's have established that elevated release of ST6Gal-1 into the blood was a component of the hepatic acute phase response (37, 38), although insight into the physiologic contribution of blood ST6Gal-1 remained largely elusive. Much is known about the catalytic specificity of this glycan-modifying enzyme, ST6Gal-1, in attachment of α2,6-linked sialic acid residues to exposed lactosaminyl-termini of glycoproteins (39, 40). However, the traditional paradigm of glycosylation was that of an intracellular process, where glycosyltransferases such as ST6Gal-1 reside within the Golgi-ER secretory apparatus and modify the transiting nascent glyco-conjugates in an individual cell-autonomous manner. In contrast, circulatory ST6Gal-1, which is secreted principally by the liver, is extracellular and operates by the novel extrinsic glycosylation mechanism that is non-cell autonomous. Extracellular ST6Gal-1 remodels marrow hematopoietic precursor cells, and in so doing, mutes the ability of the precursors to differentiate and proliferate (22).

Original studies from this laboratory uncovered a link between low circulating ST6Gal-1 and excessive *de novo* inflammatory cell production (2, 3, 5, 22). Later, we also showed that chronically raising blood-borne ST6Gal-1 activity by subcutaneous implantation of a B16 melanoma engineered to overexpress the secretory form of the enzyme was effective in diminishing production of new granulocytes by blunting the transition of GMP to GP in granulopoiesis, and this approach was effective in controlling sterile agent-induced inflammation (1). In the current report, we show that an infection-driven inflammation can also be controlled effectively by direct intravenous infusion of pure recombinant ST6Gal-1 protein, despite using a primitive rST6G formulation that is very rapidly cleared from the blood. We used a murine model of NTHI-elicited acute airway. NTHI commonly colonizes the lower airways of patients with chronic obstructive pulmonary disease (COPD) and frequently contributes to the acute exacerbations driving disease progression (41, 42). In the mouse, airway instillation of NTHI elicits an immediate acute lung inflammatory response characterized by severe neutrophil infiltration into the airway, and repeated exposure to NTHI reproduces and perpetuates many of the pathophysiologic symptoms of COPD (43). Previously we showed that a sterile LPS induced airway inflammation in mice resulted in extrinsic sialylation of pulmonary and circulating leukocytes, and the extrinsic sialylation used sialic acid precursors from activated platelets (33). We showed here that NTHI-elicited acute lung inflammation is more severe in mice with circulatory ST6Gal-1 insufficiency, as characterized by a 1.5-fold exaggeration in the already severe neutrophil infiltration into the airway. In wild-type animals, the onset of NTHI-elicited pulmonary inflammation was coincident with a specific and transient dip in ST6Gal-1 activity in circulation. This observation further supports the idea that depressed circulatory ST6Gal-1 predisposes the host for pro-inflammatory conditions and inflammatory cell production.

The detailed mechanistic links of how cell surface sialylation affects overall hematopoietic cell behavior remains to be elucidated. However, ST6Gal-1-mediated attachment of α2,6-sialic acids on β1 integrin alters cellular adhesiveness (44, 45) leading to altered cell motility (46), cancer cell differentiation and progression (46). In this study, we show that a recombinant protein corresponding to the soluble form of ST6Gal-1 (rST6G) was effective in mitigating infection-driven acute inflammation. Technical challenges associated with this approach remain. Most notably, suboptimal pharmacokinetic properties and/or enzymatic instability (Supplemental Figure 1A) of the present rST6G form resulted in undesirably rapid lost from circulation in the mouse, and rST6G remains difficult to produce in large quantities. Despite these limitations, the data show that systemic rST6G administration reduces overall marrow cellularity, dramatically decreases marrow granulocyte pool, and decreases marrow G-, GM-, and G-CFU clonogenic activities (see Figure 3). In response to NTHI challenge in the airway, rST6G intervention after the onset of localized acute inflammatory response resulted in pronounced mitigation of inflammation. While reduced granulocyte accumulation by rST6G administration was predicted by prior results, the muted release of inflammatory cytokines TNFα, IL-1β, and IL-6 in the airways was unexpected. The data showed not only a profound suppression of inflammatory cytokine release *in vivo*, but airway macrophages in culture were less able to release TNF-α and IL-6 upon exposure to heat-killed NTHI. Bone marrow derived macrophages recapitulated not only the suppression of NTHI stimulated release of TNF-α but also the augmented release of the anti-inflammatory IL-10 by rST6G treatment *ex vivo*. The data advance the idea that the ability of circulatory ST6Gal-1 to mitigate inflammation is exerted through the concerted effects of at least two distinct target mechanisms, although additional targets may be likely. The known targets are the control of hematopoietic production of inflammatory cells and suppression of inflammatory cytokines.

Together, the data show that rST6G administration has novel therapeutic potential in the management of inflammatory conditions. This approach leverages the natural function of a natively circulatory glycan-modifying enzyme, the sialyltransferase ST6Gal-1. Intervention by systemic rST6G administration elevates circulating ST6Gal-1 activity, blunting the inflammatory cytokine cascade, and suppressing *de novo* production of inflammatory cells. While not specifically examined here, blunting these components of the initial inflammatory cascade should benefit in mitigating the lasting injury such as airway remodeling and organ injury at later stages of exposure to environmental insults.

## Materials and Methods

### Animals

The *St6gal1-*dP1 and *St6gal1*-KO mice strains were in the C57BL/6J background as described previously (2). Unless otherwise stated, C57BL6/J mice between 7 and 10 weeks of age were used, and both sexes were equally represented. Roswell Park Institute of Animal Care and Use Committee (IACUC) approved maintenance of animals and all procedures used under protocol 1071M. There is no involvement of human subjects or clinical specimens; ethics committee review is not required according to the local and national guidelines.

### Recombinant ST6Gal-1 (rST6G) and Sialyltransferase Assays

rST6G is the recombinant secretory form of rat ST6Gal-1 where the catalytic domain was generated as a fusion protein encoding the following: NH_2_-signal sequence – 8x His tag – Avi tag – GFP – TEV protease cleavage site – ST6GAL1 catalytic domain – COOH (47). The construct was expressed in HEK293 cells; the recombinant protein was harvested and purified from the medium. The ST6Gal-1 catalytic domain was proteolytically released by TEV protease digestion and further purified (47, 48). Sialyltransferase assays were carried out as described previously (23).

### Acute NTHI Exposure and Recombinant ST6Gal-1 (rST6G) Treatment

A frozen glycerol stock of NTHI strain 1479 (clinical isolate from a COPD exacerbation) was streaked on chocolate-agar plates, and single colonies were grown in a liquid culture of brain–heart infusion media supplemented with 10 μg/ml hemin and 10 μg/ml β-NAD (Sigma). After 3–4 h of culture in a 37°C shaking incubator, OD_600_ was determined to dilute the required number of CFU to 2 × 10^8^ CFU/ml in PBS. Bacteria were pelleted in microcentrifuge tubes at 13,000 × g for 10 min and washed twice in PBS. To initiate acute NTHI-mediated inflammation, mice were anesthetized by isoflurane inhalation, and 50 μl (1 × 10^6^ CFU) live NTHI diluted in PBS was use for oropharyngeal instillation using a 200-μl sterile pipette tip.

rST6G was injected i.v. (750 μg/CC in PBS) 2 and 14 h after NTHI exposure. Same volume of PBS was injected to control mice. After 18 h, mice were sacrificed by injection (i.p.) of two 0.5 ml Avertin (2.5 gr 2,2,2, Tribromethanol, 5 ml 2-methyl-2-butanol in 200 ml distilled water). Bronchoalveolar lavage (BAL) was performed post euthanization by opening the thoracic cavity to expose the trachea, which was cannulated with a 22-gauge i.v. catheter. PBS (750 μL) was injected and withdrawn from the lung two times using a tuberculin syringe. For cytokine assays, BAL fluid (200 μl) or serum (50 μl) was subjected to Luminex 100 multiplex assays using a capture bead system developed by Luminex Corporation (Austin, TX, USA). For pathohistologic evaluations, lungs were excised and fixed in 10% formaldehyde in PBS, paraffin embedded, sectioned, and stained with H&E. Lung pathology was evaluated by a board certified pulmonary pathologist blinded to the identity of the slides.

### Flow Cytometry, Cell Differentials, and Bone Marrow Analysis

Flow cytometry was performed using anti-CD45 (hematopoietic cells), anti-Ly6G (neutrophils), anti-B220(B cells), anti-CD3 (T cells), anti-F4/80 (macrophage) anti-CD11c (dendritic cells) antibodies and SNA (*Sambucus nigra* lectin, Vector Laboratories, Peterborough, UK). All reagents were purchased from BioLegend (San Diego, CA). Cells were analyzed using BD LSRII flow cytometer (Becton Dickinson Immunocytometry Systems). For colony forming cell assays, marrow nucleated cells in a volume of 0.1 ml were plated in 0.9 ml of methylcellulose medium (MethoCult 3534, STEMCELL Technologies) in duplicate and placed in humidified incubator with 5% CO_2_ at 37°C. Colonies containing at least 50 cells were counted 7 days after incubation.

### *Ex vivo* Labeling of Cells and Transfer Into Recipients

Bone marrow cells were collected from hind-limbs of mice, either *St6gal1-dP1* or C57BL/6 wild-type, re-suspended in RBC lysis buffer (0.8% NH_4_Cl, 0.1 mM EDTA buffered with KHCO_3_ to pH 7.4), washed and re-suspended in phosphate-buffered saline (PBS) with 0.5% BSA or fetal bovine serum and 2 mM EDTA, and then passed through a 100-μm cell strainer (BD Biosciences). Cells were centrifuged and re-suspended in the same buffer (up to 2 × 10^8^ cells/ml), and 50 μl/ml of biotinylated antibodies (anti-cKit. anti-B220, anti-CD3, anti-TER119, anti-CD5) was added to the cell suspension. Partial neutrophil enrichment was accomplished by negative selection using magnetic microparticles according to the manufacturer's protocol (STEMCELL Technologies, Vancouver, British Columbia, Canada). More than 60% of selected cells were neutrophils as verified by flow cytometry. To apply labeling with PKH26 and PKH67 (Sigma Chemical, St Louis, MO), cells were washed in RPMI medium (without serum), and 10^7^ cells were re-suspended in 1 mL Diluent C (Sigma) and rapidly added to 1 mL of 4 μM PKH26 or PKH-67. The cells were incubated at 25°C for 5 min, terminated by the addition of 2.5% fetal calf serum. After labeling, the cells were washed twice with cold PBS and counted by hemocytometer. Differentially labeled donor cells were mixed 1:1 immediately before infusion into recipient animals. A small fraction of combined cells was labeled with neutrophil marker (anti-Ly6G antibody) and saved for measuring donor WT/dP1 neutrophil ratios. Each recipient received pooled cells consisting of 10^7^ cells intravenously from each labeled group 2 h after NTHI BAL was performed 18 h after NTHI.

### Statistics

Testing for differences between mean values was determined using either Students' *T*-Test or two-way ANOVA with post-test comparisons in Graph Pad Prism 6 software (La Jolla, CA). *p* < 0.05 is considered significant.

## Author Contributions

MN designed the research, performed the experiments, and wrote the paper. AL designed the research and performed the experiments. ZG and KM generated the recombinant enzyme. SN designed the research. PB performed histopathologic evaluations and interpreted information. YT designed the research. JL designed the research, coordinated project activities, and wrote the manuscript.

### Conflict of Interest Statement

The authors declare that the research was conducted in the absence of any commercial or financial relationships that could be construed as a potential conflict of interest.
