# Supplementary material for: Recombinant Sialyltransferase Infusion Mitigates Infection-Driven Acute Lung Inflammation
Source: Front Immunol. 2019 Feb 4;10:48. doi: 10.3389/fimmu.2019.00048 (PMC6369197; doi:10.3389/fimmu.2019.00048)
Supplement: Supplementary file 1 [file Data_Sheet_1.PDF]

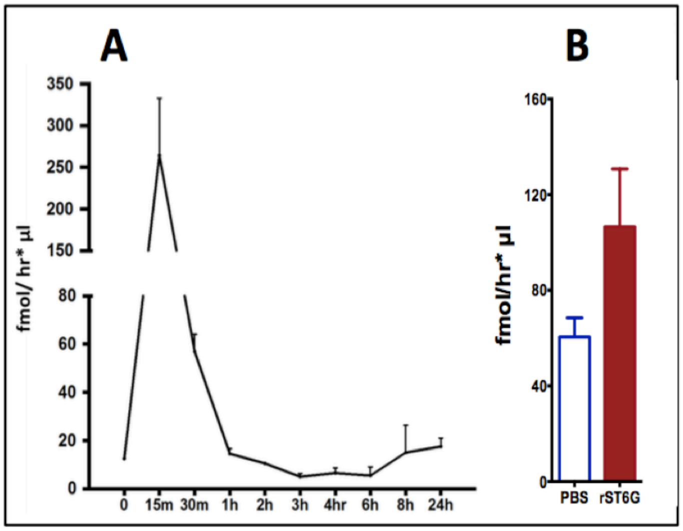

Supplemental Figure 1. Longevity of rST6G in circulation after tail vein infusion. Left Panel: Circulatory ST6Gal-1 activity was measured at different times following a single iv. tail vein injection of 300µg into resting C57BL/6 mice. Base line endogenous ST6Gal-1 activity is 12-17 fmol/hr/µl serum. **Right Panel:** rST6G, or PBS (sham) was administered i.v. at 0 and 8hrs (300µ rST6G at each interval). Blood was withdrawn at 16h after the first rST6 administration and the ST6Gal-1 activity directed at the formation of Sia α2,6 to Gal(β1,4)GlcNAc-o- Bn determined. This protocol is similar to the schematic in main Figure 4A, except that the animals did not receive a prior airway challenge with NTHI.

Supplemental Table 1A. Leukocyte accumulation in bronchoaveolar lavage 18 hours post i.t. challenge with NTHI.

	Total	Neu	Mac	DC	T	B
**Wild-type**	103 ±0.47	77 ±0.58	9.6 ±0.87	3.4 ±0.56	0.94 ±0.27	4.7 ±0.14
***St6gal1*-dP1**	149 ±19*	133 ±17*	5.6 ±28.9*	3.3 ±0.80	1.26 ±0.37	10.3 ±0.33*
***St6gal1*-KO**	170 ±30*	153 ±25*	6.5 ±25.2*	3.3 ±10.7	1.52 ±0.82	8.8 ±0.16*

Leukocyte counts ($x10^4$) in bronchoalveolar lavage fluid in mice 18 hrs after i.t. challenged with NTHI, showing total cells (Total), neutrophils (Neu), macrophage/monocytes (Mac), dendritic cells (DC), T-cells (T) and B-cells (B). * indicates values with statistically significant deviation from Wild-type values ($p < 0.01$). n=6 in all three groups.

Supplemental Table 1B. Blood deferential counts following i.v. rST6G administration.

	WBC[a]	Neut[a]	Lymph[a]	Mono[a]	Eos[a]	Baso[a]	PLT[a]	RBC[b]	HGB[c]	HCT[d]	MCV[e]	MCH[f]	MCHC[g]
**PBS**	8.8 ±1.6	0.8 ±0.2	7.3 ±1.1	0.10 ±0.0	0.30 ±0.1	0.1 ±0.0	906 ±129	9.9 ±0.6	15.3 ±0.6	49.5 ±3.9	50.7 ±2.0	15.7 ±0.2	30.9 ±1.3
**rST6G**	4.4 ±1.5	0.9 ±0.2	3.1 ±0.9	0.05 ±0.0	0.04 ±0.0	0.1 ±0.1	273.7±73	9.5 ±0.3	14.9 ±0.5	48.0 ±2.3	50.4 ±2.5	15.7 ±0.1	31.2 ±1.4
P-value	**< 0.001**	0.662	**< 0.001**	**0.006**	**0.002**	0.772	**< 0.001**	0.268	0.304	0.416	0.794	0.858	0.759

Wild type mice received a single bolus of rST6G or PBS. Blood count and differential measured at 7 hours (n= 6 for PBS and rST6G injections).
[a] $x10^6$/ml, [b] $x10^9$/ml, [c] g/dl, [d] %, [e] MCV, [f] pg, [g] g/dl

Supplementary Table 2. Histopathologic evaluation of NTHi-treated lungs between rST6G and sham treated mice.

Case \ Scores	BV Bundles [a]	Pleura [b]	Interstitial [c]	Cell composition [d] Lymphoplasmacytic	Neutrophil
rST6G 1	1	1	1	3	1
rST6G 2	1	1	1	3	1
rST6G 3	1	1	2	3	1
rST6G 4	1	0	1	2	2
rST6G 5	1	2	1	1	1
rST6G 6	1	1	1	2	2
PBS 1	1	2	2	2	2
PBS 2	2	2	2	2	2
PBS 3	2	2	1	2	2
PBS 4	2	2	2	2	2
PBS 5	2	1	2	2	2
PBS 6	2	2	2	2	2

Mice treated with NTHi as summarized in Figure 4 were subjected to histopathologic evaluation by a trained pulmonary pathologist blinded to the study groups. The scores are based on the scoring scheme as summarized below:

Lung Inflammation Scoring Scheme

I. Bronchovascular bundles
- 0- No inflammation
- 1- Minimal (average of <5 inflammatory cells per cuff thickness)
- 2- Moderate (average of 5-15 inflammatory cells per cuff thickness)
- 3- Marked (average of >15 inflammatory cells per cuff thickness and/or $\geq$ 3 peri-bronchial well-developed lymphoid follicles)

II. Pleural Inflammation
- 0- No inflammation of pleura
- 1- Mild focal inflammation without pleural thickening
- 2- Patchy thickening (expansion) of pleura by inflammatory cells and/or diffuse involvement by inflammatory cells
- 3- Diffuse pleural thickening or significant (marked) pleural inflammatory nodules

III. Interstitial Inflammation
- 0- No interstitial inflammation
- 1- Focal interstitial inflammatory infiltrate without nodules
- 2- Patchy interstitial inflammatory infiltrate, with or without occasional inflammatory nodules
- 3- Diffuse interstitial infiltrate or marked nodular aggregates of interstitial inflammatory cells

IV. Inflammatory Cell Composition (Across all tissue compartments)

Lymphoplasmacytic Inflammation
- 0- Rare to no lymphocytes or plasma cells (<1%)
- 1- 1-9% lymphocytes or plasma cells
- 2- 10-49% lymphocytes or plasma cells
- 3- >50% lymphocytes or plasma cells

Neutrophils
- 0- Rare to no neutrophils (<1%)
- 1- 1-9% neutrophils
- 2- 10-49% neutrophils
- 3- >50% neutrophils

V. Evidence of Hemorrhage

Airspace Blood and Hemosiderin: % lung involved

Bronchial Blood and Hemosiderin: # airways involved

<u>Definitions</u>
- o Focal- Limited number of discrete areas of involvement.
- o Patchy- More extensive involvement with developing confluence of adjacent areas (when appropriate).

- Diffuse- Widespread involvement with extensive confluence involving most of tissue compartment.
